# Beyond High-Speed Curing: Effects of Layer Thickness, Glycerin, and Polishing on the Surface Properties of an AFCT-Based Bulk-Fill Composite

**DOI:** 10.3390/polym18182210

**Published:** 2026-09-11

**Authors:** Ezgi Erden Kayalidere, Merve Sahin, Zeynep Hale Keles

**Affiliations:** 1 Department of Restorative Dentistry, Faculty of Dentistry, Istanbul Kent University, 34433 Istanbul, Türkiye; ezgi.kayalidere@kent.edu.tr (E.E.K.); merve.sahin@kent.edu.tr (M.S.); 2 Department of Restorative Dentistry, Faculty of Dentistry, Atlas University, 34406 Istanbul, Türkiye

**Keywords:** bulk-fill composite, photoactivation protocol, glycerin, oxygen-inhibited layer, surface roughness, microhardness, polishing

## Abstract

This in vitro study evaluated the effects of layer thickness, photoactivation protocol, glycerin application, and polishing on top-surface roughness and microhardness. Sixty specimens were prepared from bulk-fill (Tetric PowerFill) and conventional (IPS Empress Direct) composites. Bulk-fill specimens were 2 or 4 mm thick and photoactivated with a conventional (1200 mW/cm^2^, 20 s) or high-speed (3000 mW/cm^2^, 3 s) protocol, giving nominal radiant exposures of 24 and 9 J/cm^2^; conventional specimens were 2 mm thick and photoactivated using the conventional protocol. Glycerin was applied or omitted; all specimens were polymerized beneath a polyester strip, with no air-exposed group. Roughness and Vickers microhardness were measured exclusively on the top surface, before and after polishing, and analyzed using General Linear Models for repeated measures (α = 0.05). In the bulk-fill composite, polishing reduced roughness and increased microhardness, with significant polishing × glycerin interactions for both outcomes. In the conventional composite, glycerin increased microhardness and roughness before polishing. Polishing increased microhardness, and a significant polishing × glycerin interaction was observed for roughness. The 20 s protocol produced significantly higher top-surface microhardness than the 3 s protocol, although the difference was small (2.58 VHN); because irradiance and exposure time differed simultaneously, their individual contributions cannot be distinguished. Layer thickness did not significantly affect the top-surface properties evaluated; this cannot be extrapolated to deeper regions or taken as evidence of equivalence between the 2 and 4 mm increments. In the condition in which both composites were treated identically, the effects of glycerin and of polishing differed significantly between the materials for both outcomes; for roughness, the difference in glycerin effects further depended on polishing. With no air-exposed controls and no artificial aging, the findings are restricted to the immediate top-surface response under the conditions evaluated.

## 1. Introduction

With advances in dental material technology, “bulk-fill” or “mono-incremental” composites have been introduced as alternatives to conventional resin composites. These materials allow simplified clinical applications and reduced working times, as they can be placed in single increments of 4–5 mm [1,2].

To further minimize chair time, one manufacturer modified the formulation of an existing bulk-fill composite so that it can be polymerized in only 3 s with a light-curing unit delivering an irradiance of 3000 mW/cm^2^ [3,4]. The organic matrix of the pioneering material, Tetric PowerFill (Ivoclar, Schaan, Liechtenstein), contains a β-allyl sulfone reagent that activates the addition–fragmentation chain transfer (AFCT) mechanism [3,5]. This mechanism can promote a more controlled polymerization process by regulating chain growth and network formation, resulting in a more homogeneous polymer network [5,6]. Consequently, it may mitigate some of the limitations associated with ultrashort high-speed photoactivation protocols reported for previous generations of composites [7]. Bulk-fill composites also contain patented photoinitiators, one of which is a germanium-based Norrish type I photoinitiator commercially known as Ivocerin [8]. Because of its higher reactivity compared with conventional camphorquinone systems, this photoinitiator has been reported to facilitate polymerization of bulk-fill composites in increments of up to 4 mm [3,9].

Polymerization of resin composites proceeds through a free-radical reaction that is susceptible to atmospheric oxygen. Oxygen scavenges free radicals, resulting in an oxygen-inhibited superficial layer of approximately 10–200 µm, depending on the material and the curing conditions [10]. Several methods have been proposed to reduce the formation of this layer, including finishing and polishing after photoactivation, or the use of barriers, such as Mylar strips or glycerin, before photoactivation. Both Mylar strips and glycerin have been used as strategies to limit oxygen exposure during polymerization [11]. Mylar strips are readily applied to surfaces that can be directly covered, such as proximal and bucco-lingual walls, whereas glycerin can also be applied to occlusal and anatomically complex surfaces that a strip cannot adapt to.

Surface microhardness is widely used as an indirect indicator of polymerization quality and is influenced by factors such as the photoactivation protocol, layer thickness, filler characteristics, and finishing and polishing [2,12,13,14]. However, microhardness does not directly measure the degree of conversion; instead, it reflects the mechanical properties of the polymerized material and should therefore be interpreted only as an indirect surrogate of polymerization quality. Although considerable evidence is available on the polymerization behavior of bulk-fill composites, previous studies have mainly investigated photoactivation protocol, layer thickness, oxygen-inhibition control, or polishing as independent variables. In clinical practice, however, these variables are encountered simultaneously and may interact with one another, particularly in the recently introduced AFCT-based bulk-fill composites designed for ultrafast high-speed curing. Because layer thickness, photoactivation protocol, glycerin application, and subsequent polishing may jointly influence the surface characteristics of the composite, their combined effects cannot necessarily be predicted from the effect of each factor considered separately. To the best of our knowledge, no previous study has simultaneously evaluated the combined effects of ultrafast 3 s curing, glycerin application, polishing, and layer thickness on the surface properties of an AFCT-based bulk-fill composite. This represents a knowledge gap of potential clinical relevance, as these variables may collectively influence the surface characteristics of bulk-fill restorations at the time of placement and finishing. The present study addresses this question under standardized laboratory conditions and in the immediate post-polymerization state, without artificial aging.

Therefore, this study aimed to evaluate the effects of composite layer thickness, photoactivation protocol, glycerin gel application, and polishing on the top-surface roughness and microhardness of a bulk-fill resin composite. The null hypotheses were that the (H01) layer thickness (2 and 4 mm), (H02) photoactivation protocol, (H03) glycerin gel application, and (H04) polishing would have no statistically significant effects on the top-surface roughness or microhardness of the bulk-fill resin composite.

## 2. Materials and Methods

### 2.1. Study Design

**Figure 1:**
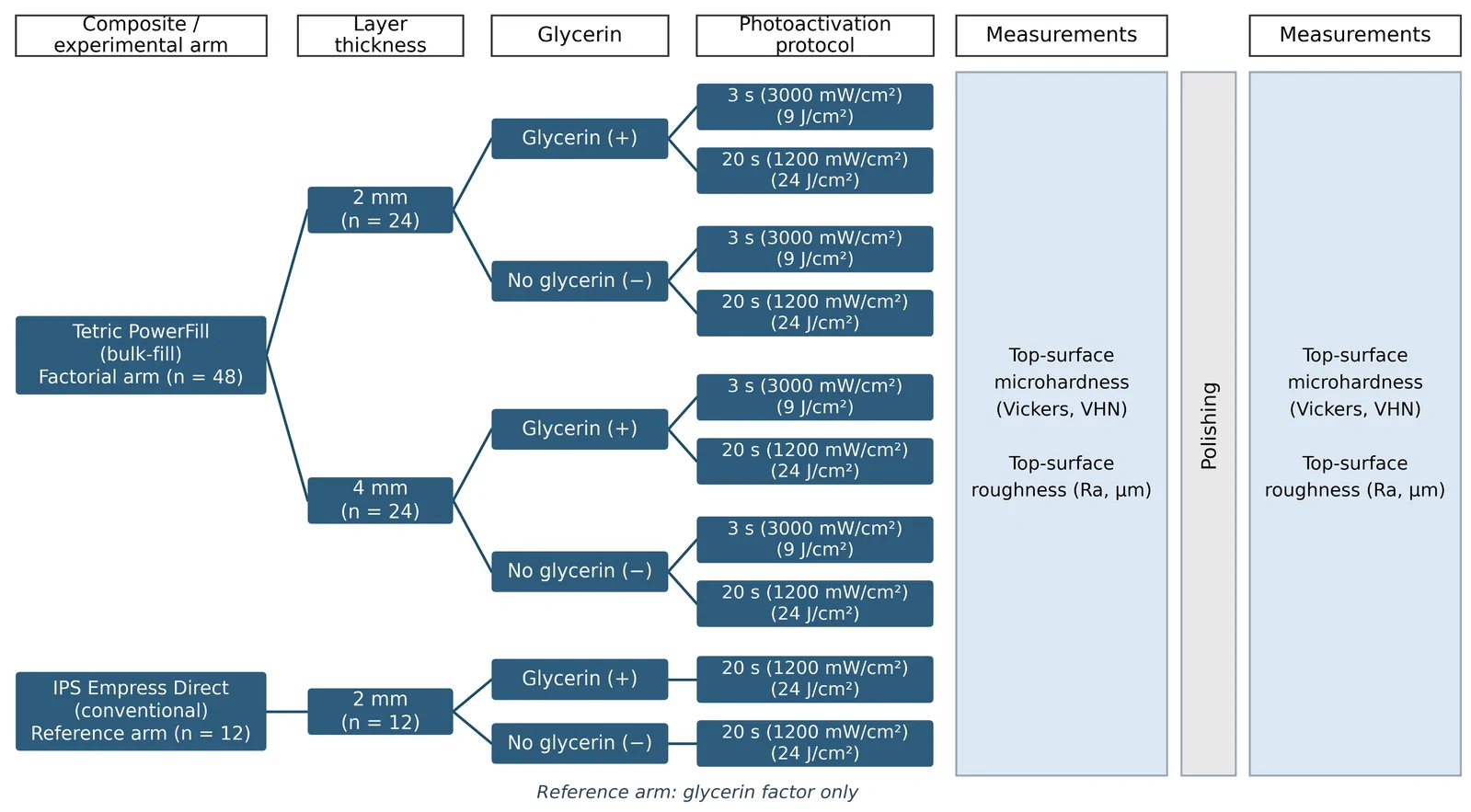
Study design and factors crossed in each experimental arm. Tetric PowerFill followed a fully crossed 2 (layer thickness) × 2 (photoactivation protocol) × 2 (glycerin application) design, whereas IPS Empress Direct served as a separate reference arm (2 mm, conventional 20 s photoactivation) with glycerin as the only between-specimen factor. Polishing was a within-specimen repeated factor. All specimens were polymerized beneath a polyester matrix strip and a 1 mm glass slide, with glycerin applied beneath the strip; no air-exposed group was included. Top-surface roughness (Ra) and Vickers microhardness (VHN) were measured before and after polishing.

The factorial part of the design was restricted to the bulk-fill composite (Tetric PowerFill, Ivoclar, Schaan, Liechtenstein), in which three between-specimen factors were fully crossed—layer thickness (2 mm/4 mm) × photoactivation protocol (high-speed 3 s/conventional 20 s) × glycerin application (yes/no)—giving eight groups of six specimens; polishing (before/after) was a within-specimen repeated factor applied to every specimen. The conventional composite (IPS Empress Direct, Ivoclar, Schaan, Liechtenstein) was included only at 2 mm and only with the conventional 20 s protocol, because a 4 mm bulk increment and the high-speed 3 s photoactivation protocol are not indicated for this material. It was therefore crossed with glycerin and polishing only and does not constitute a factorial control for layer thickness or photoactivation protocol. Statements about layer thickness and photoactivation protocol accordingly apply exclusively to the bulk-fill composite, whereas a direct comparison between the materials was possible only in the conditions common to both (2 mm, conventional 20 s photoactivation). Specimens were allocated to the corresponding experimental groups as summarized in Figure 1.

### 2.2. Sample Size Calculation

The sample size was calculated with G*Power software (version 3.1.9.7; Heinrich-Heine-Universität Düsseldorf, Düsseldorf, Germany). Because G*Power does not provide an a priori sample size procedure for the General Linear Model with repeated measures used in the present study, the calculation was based on the closest available statistical model (ANOVA: repeated measures, within–between interactions). An anticipated effect size (f = 0.39) reported in a previously published in vitro study with a comparable repeated measures design evaluating the surface properties of bulk-fill resin composites was used [2]. An α error probability of 0.05, a statistical power (1 − β) of 0.80, eight experimental bulk-fill groups (2 layer thicknesses × 2 photoactivation protocols × 2 glycerin conditions), two repeated measurements (before and after polishing), a correlation between repeated measurements of 0.50, and a nonsphericity correction (ε) of 1.0 were specified. The minimum required sample size for the eight bulk-fill groups was 32 specimens in total. To ensure equal and balanced allocation across the eight experimental conditions, six specimens were included in each group, giving 48 bulk-fill specimens. The design also included two conventional composite groups. As layer thickness and photoactivation protocol were not experimental variables for the conventional composite, these groups were not included in the a priori sample size calculation and were analyzed separately. To maintain consistency with the bulk-fill groups, the same group size (*n* = 6) was used for each conventional composite group, giving 12 additional specimens. Accordingly, a total of 60 specimens were included in the study.

### 2.3. Specimen Preparation

Two resin composites were evaluated: a bulk-fill composite (Tetric PowerFill) and a conventional nanohybrid composite (IPS Empress Direct). Detailed information on the materials is provided in Table 1. A total of 60 disk-shaped specimens (6 mm in diameter) were fabricated in a standardized cylindrical mold. For the bulk-fill composite, 48 specimens were prepared and equally divided between two thicknesses: 24 specimens of 2 mm and 24 specimens of 4 mm. For the conventional composite, 12 specimens were fabricated at a thickness of 2 mm, following the manufacturer’s instructions. All procedures were performed by a single operator to minimize variability.

During specimen preparation, a polyester matrix strip (Hawe Polyester Matrix Strip; Kerr, Bioggio, Switzerland) and a 1 mm thick glass slide were placed over the composite to standardize specimen thickness, surface flatness, and the position of the specimen surface relative to the light-curing tip. The polyester matrix strip and glass slide were applied identically in all experimental groups. This arrangement was used in every group so that no specimen surface was polymerized in direct contact with atmospheric air. The design therefore contrasts a polyester strip alone with a polyester strip combined with glycerin and does not permit the independent effects of the strip and glycerin, or their combined effects on oxygen inhibition, to be separated.

In the glycerin groups, after the composite had been placed in the mold, glycerin gel (Miraderm Pharma, Istanbul, Türkiye) was applied onto the uncured composite with a brush in a single direction. Immediately thereafter, the matrix strip and glass slide were positioned over the gel-covered surface and gentle pressure was applied. Polymerization was performed with a light-emitting diode (LED)-curing unit (Bluephase PowerCure, Ivoclar, Schaan, Liechtenstein) with a wavelength range of 390–500 nm. This emission spectrum encompasses the absorption ranges of both camphorquinone and the germanium-based photoinitiator (Ivocerin) present in the tested materials, thereby ensuring compatibility with their respective photoinitiator systems. The irradiance output of the curing unit was verified with the radiometer integrated into the charging base immediately before each specimen was polymerized. The emission spectrum was not independently measured and was taken from the manufacturer’s specifications.

Specimens made from the conventional composite were polymerized exclusively with the conventional photoactivation protocol (1200 mW/cm^2^ for 20 s; nominal radiant exposure 24 J/cm^2^). For the bulk-fill composite, specimens were divided into two curing pathways: half were polymerized with the conventional protocol and the remainder with the high-speed protocol (3000 mW/cm^2^ for 3 s; nominal radiant exposure 9 J/cm^2^). During photoactivation, the polyester matrix strip and the 1 mm thick glass slide remained in place over the specimen. The irradiance output of the LED unit was verified with its integrated radiometer immediately before each curing cycle, with the light tip positioned directly on the radiometer sensor without interposition of the glass slide or polyester matrix strip. After polymerization, any remaining glycerin residue was thoroughly rinsed off with an air–water spray and the specimens were gently dried. All specimens were then stored in an incubator at 37 °C for 24 h to allow post-cure polymerization.

### 2.4. Finishing and Polishing

Microhardness and surface roughness were measured on all specimens both before and after polishing. The specimens were polished under dry conditions with aluminum-oxide-coated disks (Sof-Lex; 3M ESPE, St. Paul, MN, USA). Coarse- and medium-grit disks were applied at 10,000 rpm, followed by fine- and superfine-grit disks at 30,000 rpm, each polishing step lasting 15 s. Following the manufacturer’s instructions, the specimen surfaces were thoroughly rinsed with water and dried after each disk step to remove residual abrasive particles. A new polishing disk was used for each specimen to avoid abrasive wear and to ensure consistent polishing efficiency.

### 2.5. Surface Roughness and Microhardness Measurements

Surface roughness and microhardness were measured exclusively on the top surfaces of the specimens before and after polishing. For each measurement instance, measurements were obtained from the same standardized region of the top surface of each specimen. Surface roughness was recorded before microhardness so that no profilometer trace passed over a Vickers indentation.

Surface microhardness was measured with a digital microhardness tester (Falcon 400, Innovatest, Maastricht, The Netherlands). A Vickers diamond indenter was applied to the top surface of each specimen with a 100 g load for 10 s. Three indentations were made per specimen at separate locations within the central region of the surface and away from the margins. The indentations were examined at 20× magnification, and the diagonals of each indentation were measured individually. The mean of the three indentations was recorded as a specimen-level Vickers hardness number (VHN).

Surface roughness was measured with a contact stylus profilometer (Mitutoyo Surftest SJ-410, Mitutoyo Corporation, Kawasaki, Japan) fitted with a diamond stylus with a 2 µm tip radius and a 60° included angle, using a measuring force of 0.75 mN and a traverse speed of 0.5 mm/s. A Gaussian profile filter was applied with a sampling length (cut-off, λc) of 0.25 mm; the total traverse length was 2 mm, of which an evaluation length of 1.25 mm (five sampling lengths) was assessed. This cut-off was selected because the 6 mm specimen diameter did not allow a longer sampling length to be traversed within the flat central area of the disk and was kept constant for all specimens and measurement occasions. Five traces were recorded at different locations within the central region of the top surface of each specimen using the same measurement procedure. The arithmetic mean of the five traces was recorded as a specimen-level mean surface roughness (Ra), expressed in micrometers (µm). The individual specimen, rather than the individual indentation or profilometric trace, was the experimental unit.

### 2.6. Statistical Analysis

Statistical analyses were performed with NCSS (Number Cruncher Statistical System) 2020 Statistical Software (NCSS, LLC, Kaysville, UT, USA). To account for the repeated measurements obtained before and after polishing on the same specimens, General Linear Models for repeated measures (GLMs) were constructed separately for each material and outcome variable (surface roughness and microhardness). Polishing was specified as the within-specimen (repeated) factor in all models. For the bulk-fill composite, glycerin application, layer thickness, and photoactivation protocol were included as between-specimen factors, whereas for the conventional composite, glycerin application was the only between-specimen factor. The experimental unit in all models was the individual specimen, and each specimen contributed exactly two observations, one before and one after polishing; repeated readings within a specimen were never treated as independent replicates.

Categorical variables were dummy coded to estimate regression coefficients (β) and their corresponding 95% confidence intervals (CIs). For the bulk-fill composite, the initial model was a full factorial model, containing the four main effects (polishing, glycerin, layer thickness, and photoactivation protocol), all six two-way interactions between them, all four three-way interactions, and the four-way interaction. For the conventional composite, in which only glycerin and polishing were varied, the initial model contained both the main effects and their interactions. All main effects were retained regardless of statistical significance. Interaction terms were then reduced sequentially using backward elimination, beginning with the highest-order terms, with the model refitted after each step. Non-significant higher-order interaction terms were removed first, followed by non-significant lower-order interaction terms, until the final reduced model was obtained. The polishing × glycerin interaction was the only interaction retained in the final models, being statistically significant for both outcomes in the bulk-fill composite and for surface roughness in the conventional composite. The initial full factorial models, including all interaction terms subsequently removed during model reduction, are reported in Table S1 to provide a transparent account of the interaction analyses. For statistically significant interactions retained in the final material-specific models, simple-effects analyses were additionally performed to estimate the effect of glycerin separately before and after polishing and the effect of polishing separately with and without glycerin, because these contrasts are more directly interpretable than the coefficient of the interaction term.

Because a difference in statistical significance between two separately fitted models does not itself demonstrate that an effect differs between materials, an additional analysis was performed using only the experimental conditions common to both composites (2 mm thickness and conventional 20 s photoactivation). In this matched subset, material and glycerin application were specified as between-specimen factors and polishing as the within-specimen repeated factor. The model included the main effects of material, glycerin, and polishing, and all corresponding two- and three-way interactions; the material × glycerin, material × polishing, and material × glycerin × polishing interaction terms provided formal tests of whether the corresponding effects differed between materials under these common experimental conditions. No interaction terms were removed from this additional model, as its purpose was to test the prespecified interactions involving material directly. This analysis comprised 24 specimens, 12 per material, each contributing measurements before and after polishing, with the estimates reported in Table S2.

Model assumptions were assessed by evaluating the normality of residuals using the Shapiro–Wilk test and the homogeneity of variances using Levene’s test. Regression coefficients (β), 95% CIs, and *p*-values are reported. The level of statistical significance was set at *p* < 0.05.

## 3. Results

Descriptive statistics for surface roughness and microhardness are presented in Table 2 and Table 3, respectively. Table 4, Table 5, Table 6 and Table 7 summarize the factors influencing the surface roughness and microhardness of bulk-fill composite specimens (Table 4 and Table 6) and conventional composite specimens (Table 5 and Table 7).

### 3.1. Surface Roughness

For the Tetric PowerFill group, polishing was associated with a significant reduction in surface roughness (β = −0.47 µm; *p* < 0.001) (Table 4). Layer thickness had no statistically significant effect on surface roughness (β = 0.03 µm; *p* = 0.348). Similarly, the photoactivation protocol had no statistically significant influence on surface roughness (β = 0.05 µm; *p* = 0.112). Glycerin increased surface roughness (β = 0.69 µm; *p* < 0.001), but this effect was observed only in unpolished specimens. A significant negative polishing × glycerin interaction was found (β = −0.66 µm; *p* < 0.001), with polishing producing a greater reduction in surface roughness in the glycerin-treated subgroups than in the non-glycerin subgroups. Because the polishing × glycerin interaction was significant, the glycerin effect was estimated separately at each level of polishing; before polishing, glycerin increased roughness by 0.69 µm (95% CI 0.59 to 0.78; *p* < 0.001), whereas after polishing, the difference was 0.02 µm (95% CI −0.07 to 0.12; *p* = 0.626). Conversely, polishing reduced roughness by 0.47 µm (95% CI 0.38 to 0.57; *p* < 0.001) in specimens cured without glycerin and by 1.14 µm (95% CI 1.04 to 1.23; *p* < 0.001) in glycerin-treated specimens. After polishing, no statistically significant difference in roughness remained between the glycerin and non-glycerin conditions.

For the IPS Empress Direct group, glycerin application significantly increased roughness (β = 1.69 µm; *p* < 0.001), whereas polishing did not produce a significant reduction (β = 0.03 µm; *p* = 0.824). The polishing × glycerin interaction was, however, significant (β = −1.64 µm; *p* < 0.001) (Table 5). Expressed as simple effects, glycerin increased roughness by 1.69 µm (95% CI 1.41 to 1.97; *p* < 0.001) before polishing and by 0.05 µm (95% CI −0.23 to 0.32; *p* = 0.728) after polishing; polishing changed roughness by 0.03 µm (95% CI −0.26 to 0.32; *p* = 0.824) without glycerin and reduced it by 1.61 µm (95% CI 1.34 to 1.89; *p* < 0.001) with glycerin. After polishing, the mean Ra of every group in the study lay between 0.10 and 0.15 µm, the largest difference between any two polished groups being 0.05 µm.

### 3.2. Surface Microhardness

For the Tetric PowerFill group, polishing had a significant positive effect on microhardness (β = 28.97 VHN; *p* < 0.001). Glycerin application (β = 1.47 VHN; *p* = 0.301) and layer thickness (β = 1.51 VHN; *p* = 0.135) had no significant effect. Compared with the 3 s high-speed photoactivation protocol (reference category), the conventional 20 s photoactivation protocol was associated with significantly higher top-surface microhardness (β = 2.58 VHN; *p* = 0.012). Among the interaction terms, only the polishing × glycerin interaction was significant (β = −5.52 VHN; *p* = 0.007), indicating that the polishing-induced increase in microhardness was smaller in the glycerin-treated subgroups than in the non-glycerin subgroups (Table 6). Expressed as simple effects, glycerin did not significantly change microhardness before polishing (β = 1.47 VHN; 95% CI −1.33 to 4.27; *p* = 0.301) but was associated with lower microhardness after polishing (β = −4.06 VHN; 95% CI −6.86 to −1.25; *p* = 0.005); polishing increased microhardness by 28.97 VHN (95% CI 26.02 to 31.91; *p* < 0.001) without glycerin and by 23.44 VHN (95% CI 20.64 to 26.25; *p* < 0.001) with glycerin.

For the IPS Empress Direct group, both polishing (β = 40.31 VHN; *p* < 0.001) and glycerin application (β = 3.31 VHN; *p* = 0.016) significantly increased microhardness (Table 7). The polishing × glycerin interaction was not significant for this material (β = 1.20 VHN; 95% CI −4.79 to 7.18; *p* = 0.666) and was therefore not retained.

### 3.3. Interaction Analyses and Between-Material Comparison

The initial full factorial models, including every interaction term that was subsequently removed, are given in Table S1. In the bulk-fill composite, polishing × glycerin was significant for both outcomes in the initial model (roughness, *p* < 0.001; microhardness, *p* = 0.002). Glycerin × layer thickness was significant for roughness (*p* = 0.007) but not for microhardness (*p* = 0.914), and none of the remaining two-way interactions were significant for either outcome (roughness, *p* ≥ 0.119; microhardness, *p* ≥ 0.256). Two higher-order terms had *p*-values below 0.05 in the initial models—glycerin × layer thickness × photoactivation protocol for roughness (*p* = 0.041) and polishing × glycerin × layer thickness for microhardness (*p* = 0.015). Because the model was refitted after each elimination step, the statistical significance of individual terms could change as other terms were removed, and neither was significant at the step at which it was removed (Table S3). The same applies to glycerin × layer thickness, which was significant for roughness in the initial model (*p* = 0.007) but not once the higher-order terms had been removed (*p* = 0.198). Polishing × glycerin × layer thickness for roughness had *p* = 0.051 in the initial model; it was carried forward to the intermediate step and removed at the subsequent step. Retaining it in the final model changes neither the direction nor the statistical significance of any effect reported in Table 4.

Between-material differences were formally tested in the matched subset of specimens representing the experimental conditions common to both composites (2 mm increment, 20 s conventional protocol; 24 specimens, 12 per material, each measured before and after polishing; Table S2). For surface roughness, the glycerin × material interaction was significant (β = 0.49 µm; 95% CI 0.25 to 0.74; *p* < 0.001), as were the polishing × material (β = 0.46 µm; 95% CI 0.12 to 0.79; *p* = 0.011) and polishing × glycerin × material (β = −0.93 µm; 95% CI −1.41 to −0.45; *p* < 0.001) interactions. Thus, between-material differences in the glycerin effect and in the response to polishing were detected, with the significant three-way interaction indicating that the between-material difference in the glycerin effect further depended on polishing status. For microhardness, the glycerin × material interaction was also significant (β = 6.10 VHN; 95% CI 1.91 to 10.29; *p* = 0.007), as was the polishing × material interaction (β = 7.40 VHN; 95% CI 1.55 to 13.25; *p* = 0.016), whereas the polishing × glycerin × material interaction was not significant (β = 7.60 VHN; 95% CI −0.67 to 15.88; *p* = 0.070). Thus, the overall microhardness response to glycerin differed between the two composites, but there was no statistically significant evidence that this between-material difference additionally depended on polishing status.

## 4. Discussion

Based on the results, the first null hypothesis (H01) was not rejected, as layer thickness did not significantly affect either top-surface roughness or top-surface microhardness in the bulk-fill composite. Because no measurements were performed on the bottom surface or within the bulk of the material, this finding is restricted to the evaluated top surface. Failure to reject the null hypothesis does not provide evidence that the two layer thicknesses are equivalent. Because no equivalence margin was prespecified and no equivalence analysis was performed, the present findings cannot establish equivalence or comparability between the 2 and 4 mm increments. The second null hypothesis (H02) was rejected for top-surface microhardness, as the conventional 20 s photoactivation protocol resulted in significantly higher values than the 3 s high-speed protocol, but was not rejected for top-surface roughness. The third null hypothesis (H03) was rejected for top-surface roughness, which was significantly increased by glycerin application. For top-surface microhardness in the bulk-fill composite, glycerin had no significant overall effect, but its effect depended on the polishing status, with microhardness being significantly lower in the glycerin-treated specimens after polishing; H03 was therefore also rejected for this outcome. The fourth null hypothesis (H04) was rejected for both outcomes, as polishing significantly reduced top-surface roughness and increased top-surface microhardness in the bulk-fill composite.

Findings in the literature regarding the influence of glycerin on surface properties remain inconsistent, and no consensus exists on its optimal use in combination with finishing and polishing procedures [2]. In the present study, glycerin was applied with the intention of further limiting contact between atmospheric oxygen and the composite surface, and thus of modifying the characteristics of the superficial resin layer. Although Lassila et al. and Strnad et al. suggested that a Mylar strip controls the oxygen-inhibited layer by blocking contact between the material and oxygen, it has been reported that this method can trap bubbles during placement and may not eliminate formation of the oxygen-inhibited layer [11,15]. Glycerin was therefore selected as an additional strategy to minimize oxygen exposure, similar to the method used by Gaviria-Martínez et al. [1].

The initial increase in surface roughness observed after glycerin application, before polishing, may be attributable to the physical and chemical behaviors of the glycerin layer beneath the polyester matrix strip during specimen preparation. When applied onto the uncured composite, the hydrophilic glycerin gel may physically alter the precise adaptation and flatness of the polyester matrix strip under pressure, and the flow of the uncured material beneath it, leading to subtle superficial irregularities before photoactivation. The inconsistent findings reported in previous studies may similarly reflect differences in material composition, filler characteristics, resin viscosity, and experimental protocols rather than a uniform effect of glycerin itself [1,13]. This explanation is, however, inferred from the roughness values themselves. No surface morphological characterization, such as scanning electron or atomic force microscopy or profile imaging, was performed, so the proposed physical interaction between the glycerin layer, the composite surface, and the polyester matrix strip remains a hypothesis rather than an observation. Morphological evaluation of the cured surface would be required to confirm or refute it.

The significant negative polishing × glycerin interaction observed for surface roughness in both composites suggests that glycerin application modified the response of the composite surface to subsequent polishing. One possible explanation is that glycerin, by limiting oxygen diffusion during polymerization, reduced the formation of the oxygen-inhibited superficial resin layer. As a result, polishing may have acted on a surface with different superficial characteristics from that of conventionally polymerized specimens, leading to a greater reduction in surface roughness. Although glycerin application significantly increased surface roughness before polishing, polishing brought the mean surface roughness of all experimental groups to values below 0.2 µm, a value frequently cited in the literature in relation to plaque accumulation [16]. This mechanistic account should be read as a hypothesis rather than as a demonstrated mechanism: neither the thickness of the oxygen-inhibited layer nor the degree of conversion was measured in the present study, and the design contains no air-exposed group against which the oxygen-inhibiting role of glycerin could be isolated. What the data can establish is the interaction itself—that the surface response to polishing differed according to whether glycerin had been applied—and not its cause.

It is equally important to distinguish statistical significance from clinical relevance. The 0.2 µm value cited above should be regarded as a reference value rather than an absolute clinical threshold, and statistical significance should therefore be interpreted together with the magnitude of the observed differences. After polishing, mean Ra values across all groups ranged from 0.10 to 0.15 µm, with a maximum between-group difference of approximately 0.05 µm. In contrast, before polishing, glycerin was associated with increases in mean Ra of 0.69 µm in the bulk-fill composite and 1.69 µm in the conventional composite. Thus, although statistically significant effects were observed, the small residual differences after polishing may have limited clinical relevance; however, their clinical significance cannot be established from the present in vitro data.

A similar distinction applies to microhardness. The difference between the two photoactivation protocols in the bulk-fill composite (2.58 VHN) and the effect associated with glycerin in the conventional composite (3.31 VHN) were modest compared with the changes associated with polishing (28.97 and 40.31 VHN, respectively). Because no established threshold defines a clinically meaningful difference in Vickers microhardness in this context, these statistically significant differences should not be assumed to represent clinically meaningful differences in material performance.

In the present study, the Sof-Lex system was used as the finishing and polishing procedure applied after the baseline measurements, and was selected for its reported effectiveness in reducing surface roughness [2,17,18]. Successful polymerization of resin-based composites is associated with a high surface hardness and depends on numerous factors, including the photoactivation protocol, application parameters, and the chemical composition and physical properties of the material. Surface microhardness is widely used as an indirect indicator of polymerization quality, and higher degrees of monomer-to-polymer conversion have been associated with higher hardness values [2]. It remains, however, an indirect measure, as hardness reflects the mechanical response of the polymerized material and cannot by itself substantiate claims about monomer conversion, cross-link density, network homogeneity, or residual monomer release, none of which were measured in the present study.

In the current study, polishing significantly increased microhardness in both composites. Similarly, Keleş and Üçüncü reported significantly higher microhardness values after polishing across several resin composites, including Tetric PowerFill, supporting the influence of surface finishing on microhardness measurements [19]. Previous studies have suggested that this increase may be related to the removal of the superficial resin-rich composite layer, exposing an underlying layer with a higher surface hardness [10]. However, the amount of material removed was not quantified in the present study and therefore cannot be inferred directly from our experimental conditions. One possible explanation for the observed increase in microhardness is the removal of the superficial resin-rich layer and the exposure of an underlying region with a higher surface hardness. This subsurface region is less affected by atmospheric oxygen and has been reported to experience a greater temperature rise during polymerization than the outermost surface, which may favor a higher degree of conversion [20]. For microhardness, the polishing × glycerin interaction was statistically significant in the Tetric PowerFill group, suggesting that glycerin application influenced the response of the superficial layer of this material to subsequent polishing. One possible explanation is that, in the absence of glycerin, polishing removed a greater proportion of the soft oxygen-inhibited superficial resin layer, resulting in a larger increase in surface microhardness. In contrast, when glycerin was applied beneath the polyester matrix strip, oxygen inhibition may have been reduced during polymerization, resulting in a better-polymerized superficial layer before polishing and, consequently, a smaller relative increase in microhardness after the same polishing procedure. The significant interaction therefore suggests that the microhardness-enhancing effect of polishing was attenuated when glycerin was used, possibly because reduced oxygen inhibition had already modified the superficial polymerization dynamics and diminished the additional hardening effect associated with polishing. As the degree of conversion was not measured directly in the present study, these explanations should be considered inferential. For the IPS Empress Direct group, in contrast, glycerin application as a main factor significantly increased microhardness. This positive effect may be related to the ability of glycerin to limit oxygen inhibition during photoactivation, potentially promoting more effective superficial polymerization. A polishing × glycerin interaction was not retained for microhardness in IPS Empress Direct. In the matched-subset analysis, the glycerin × material interaction was significant (*p* = 0.007), as was the polishing × material interaction (*p* = 0.016), so the difference between the two composites in the microhardness response to glycerin is supported by a direct test rather than by a comparison of significance across separate models. The three-way polishing × glycerin × material interaction was not significant (*p* = 0.070), so the present data do not establish that this material difference varies with polishing status. These differences may reflect differences in resin matrix composition, photoinitiator systems, polymerization kinetics, and filler characteristics. These explanations likewise remain hypothetical, as the cross-link density was not measured.

In the present study, Tetric PowerFill specimens were polymerized with either a high-speed protocol (3000 mW/cm^2^ for 3 s) or a conventional protocol (1200 mW/cm^2^ for 20 s). The conventional photoactivation protocol resulted in significantly higher microhardness values. In a previous study, materials polymerized for 20 s via light exhibited considerably higher degrees of conversion on the top surface than those cured for only 3 s [21]. It has been reported that shorter protocols applied with a high irradiance (e.g., 3 s at 3000 mW/cm^2^) do not always provide sufficient polymerization, and that the total energy dose is a critical determinant of polymer network formation. Although the 3 s high-speed protocol initiates polymerization rapidly, protocols delivering a lower nominal radiant exposure have been reported to yield a lower degree of conversion and greater internal porosity [22]; neither were measured here. In contrast, the conventional protocol with moderate irradiance (≈1200 mW/cm^2^) delivers a higher nominal radiant exposure (24 J/cm^2^), which in those reports was associated with a greater degree of conversion and a more developed polymer network, particularly in deeper regions. Rapid polymerization may also cause premature radical depletion and an early plateau in the polymerization process, especially in materials containing highly reactive photoinitiators such as TPO and Ivocerin [22]. The higher top-surface microhardness obtained with the conventional photoactivation protocol is therefore consistent with previous studies, although the observed difference cannot be attributed independently to any single photoactivation parameter. Because the present study evaluated only top-surface microhardness and did not directly assess degree of conversion, depth of cure, or polymerization throughout the restoration thickness, this interpretation should be considered indirect. It must be stressed that the two protocols differed simultaneously in irradiance (3000 vs. 1200 mW/cm^2^) and exposure time (3 vs. 20 s), resulting in different nominal radiant exposures (9 vs. 24 J/cm^2^). Consequently, the individual contributions of irradiance and exposure time cannot be disentangled in the present design. Separating them would require a design in which irradiance and exposure time are systematically and independently manipulated. This uncertainty is compounded by the fact that the effective irradiance reaching the specimen surface was not determined: irradiance was verified at the light tip with the integrated radiometer, without the interposed polyester matrix strip and 1 mm glass slide and without spectroradiometric measurement of the beam profile, so that the potential attenuation by these interposed materials was not quantified and the actual irradiance and radiant exposure reaching the composite surface remain unknown. All radiant-exposure values are therefore reported as nominal.

Layer thickness alone did not significantly affect top-surface microhardness. This finding must be interpreted in light of the experimental design: irrespective of layer thickness, the top surface of each specimen was positioned at the same distance from the light tip and was therefore exposed to essentially the same irradiation conditions. Consequently, the absence of a significant difference in top-surface microhardness between the 2 and 4 mm increments does not permit assessment of the effect of layer thickness on polymerization at depth, where thickness-related differences may be more apparent. The present findings should therefore be restricted to the evaluated top surface and should not be extrapolated to the entire restoration thickness or interpreted as evidence of comparable depth of cure. Although previous studies have reported adequate top-surface hardness in bulk-fill composites, polymerization efficiency may differ in deeper regions [23,24,25]. Future studies incorporating bottom-surface microhardness, bottom-to-top hardness ratios, or direct degree-of-conversion measurements at different depths are required to clarify the influence of layer thickness on polymerization throughout the restoration.

This study has several limitations. First, microhardness and roughness were evaluated only on the top surface of the specimens, and polymerization at deeper regions was not assessed directly. This limitation is particularly relevant to the layer thickness comparison because the top surfaces of the 2 and 4 mm increments were exposed under essentially the same irradiation conditions. Therefore, the absence of a significant layer thickness effect on the evaluated top-surface properties should not be interpreted as evidence of comparable polymerization throughout the restoration thickness.

Second, oxygen inhibition was not isolated as an experimental variable. All specimens were polymerized beneath a polyester matrix strip and glass slide, with no air-exposed condition, and glycerin was applied beneath the strip in the corresponding groups. Because the strip itself limits contact with atmospheric oxygen, the two conditions may have differed in the extent of oxygen exclusion rather than in its presence or absence, and the design does not allow the additional effect of glycerin on oxygen inhibition to be isolated from the effect of the strip or from potential physical effects of glycerin at the strip–composite interface. No surface morphological characterization was performed, and neither the thickness of the oxygen-inhibited layer nor the degree of conversion was directly measured. Accordingly, the observed glycerin-associated differences should be interpreted as effects of the additional application of glycerin under these standardized conditions rather than as evidence of an isolated oxygen-blocking mechanism, and the proposed explanations involving oxygen inhibition, surface morphology, and polymer-network formation remain hypothetical. Future studies incorporating appropriate air-exposed, strip-only, glycerin-only, and combined conditions, together with direct characterizations of the oxygen-inhibited layer and degrees of conversion, are needed to distinguish these mechanisms.

Third, the conventional composite was not subjected to the same full factorial conditions as the bulk-fill composite and therefore represents a separate reference arm rather than a factorial control for layer thickness or photoactivation protocol. Direct between-material comparisons were restricted to the common 2 mm/20 s condition and should not be generalized to the 4 mm or high-speed photoactivation conditions.

Fourth, irradiance was verified at the light-curing unit tip rather than at the specimen surface through the polyester matrix strip and glass slide. Potential attenuation by these interposed materials was not quantified; therefore, the actual irradiance and radiant exposure reaching the composite surface remain unknown. In addition, the a priori sample-size calculation was based primarily on main effects and the within × between interaction and was not designed to ensure adequate power for interactions among the between-specimen factors. With six specimens per cell, the power to detect such interactions was therefore limited, and non-significant interactions should be interpreted cautiously rather than as evidence of the absence of an interaction.

Finally, the study was conducted under standardized laboratory conditions and evaluated only one finishing and polishing system, one bulk-fill composite, one conventional composite, and two specific photoactivation protocols. Intraoral conditions were not reproduced, and no artificial aging procedures, such as thermocycling, water aging, toothbrushing simulation, or masticatory simulation, were performed. The findings should therefore be restricted to the materials and protocols tested in the immediate post-polymerization state and should not be extrapolated to long-term clinical behavior.

## 5. Conclusions

Within the limitations of this in vitro study and for the top surface of the specimens, glycerin application increased surface roughness in both resin composites. Under the common 2 mm/20 s condition, the effects of glycerin and polishing differed between the two composites for both outcomes; for surface roughness, the between-material difference in the glycerin effect further depended on polishing status. The finishing and polishing procedure minimized the glycerin-associated increase in roughness, resulting in mean Ra values of 0.10–0.15 µm across all groups.

In the bulk-fill composite, the conventional 20 s photoactivation protocol produced higher top-surface microhardness than the 3 s high-speed protocol. Because these protocols differed simultaneously in irradiance and exposure time and the effective irradiance at the specimen surface was not measured, this difference cannot be attributed to any single photoactivation parameter. Layer thickness did not significantly influence the evaluated top-surface properties; this finding should not be extrapolated to deeper regions or interpreted as evidence of comparable depth of cure. The mechanisms proposed to explain the polishing × glycerin interaction remain hypothetical because neither the oxygen-inhibited layer nor the degree of conversion was directly evaluated. These findings are restricted to the immediate post-polymerization state under standardized laboratory conditions and cannot be extrapolated to long-term material behavior. Further studies incorporating direct polymerization measurements, assessment at different depths, appropriate oxygen-exposure controls, and artificial aging are warranted.

## Figures and Tables

**Table 1:** Materials used in the study.

Material	Composition	Manufacturer
Tetric PowerFill	Organic matrix: copolymer, Bis-GMA, Bis-PMA, UDMA, Bis-EMA. Inorganic fillers: barium glass, Si–Zr mixed oxide, ytterbium trifluoride (53–54 vol%); inorganic filler particle size 40 nm–3 µm. Photoinitiators: Ivocerin (germanium-based) and camphorquinone (CQ).	Ivoclar, Schaan, Liechtenstein
IPS Empress Direct	Organic matrix: UDMA, Bis-GMA, TEGDMA. Inorganic fillers: barium glass 0.4 µm, prepolymer 1–10 µm, ytterbium trifluoride 100 nm (52–59 vol%). Photoinitiators: camphorquinone (CQ) and amine co-initiators.	Ivoclar, Schaan, Liechtenstein
Glycerin gel	Pure glycerin.	Miraderm Pharma, Istanbul, Türkiye
Sof-Lex system	Aluminum-oxide-coated disks: coarse 60 µm, medium 29 µm, fine 14 µm, superfine 5 µm.	3M ESPE, St. Paul, MN, USA

Bis-GMA, bisphenol A-glycidyl methacrylate; Bis-PMA, bisphenol A polyethylene glycol dimethacrylate; Bis-EMA, ethoxylated bisphenol A dimethacrylate; UDMA, urethane dimethacrylate; TEGDMA, triethylene glycol dimethacrylate.

**Table 2:** Descriptive statistics (mean ± SD) of surface roughness (Ra, µm) for the bulk-fill and conventional composites, stratified by layer thickness, photoactivation protocol, glycerin application, and polishing condition (*n* = 6 per group).

Composite	Thickness	Glycerin	Photoactivation Protocol	Before Polishing Mean ± SD	After Polishing Mean ± SD
Tetric PowerFill	2 mm	+	3 s	1.27 ± 0.33	0.14 ± 0.03
Tetric PowerFill	2 mm	−	3 s	0.46 ± 0.19	0.11 ± 0.02
Tetric PowerFill	2 mm	+	20 s	1.29 ± 0.28	0.14 ± 0.07
Tetric PowerFill	2 mm	−	20 s	0.55 ± 0.18	0.12 ± 0.03
Tetric PowerFill	4 mm	+	3 s	1.11 ± 0.22	0.14 ± 0.04
Tetric PowerFill	4 mm	−	3 s	0.66 ± 0.16	0.11 ± 0.02
Tetric PowerFill	4 mm	+	20 s	1.42 ± 0.16	0.12 ± 0.02
Tetric PowerFill	4 mm	−	20 s	0.66 ± 0.21	0.11 ± 0.03
IPS Empress Direct	2 mm	+	20 s	1.77 ± 0.46	0.15 ± 0.01
IPS Empress Direct	2 mm	−	20 s	0.07 ± 0.02	0.10 ± 0.03

+, glycerin applied; −, no glycerin applied. SD, standard deviation; Ra, arithmetic mean surface roughness.

**Table 3:** Descriptive statistics (mean ± SD) of surface microhardness (VHNs) for the bulk-fill and conventional resin composites, stratified by layer thickness, photoactivation protocol, glycerin application, and polishing condition (*n* = 6 per group).

Composite	Thickness	Glycerin	Photoactivation Protocol	Before Polishing Mean ± SD	After Polishing Mean ± SD	Change After Polishing Mean ± SD
Tetric PowerFill	2 mm	+	3 s	41.43 ± 1.81	56.97 ± 3.62	15.54 ± 3.17
Tetric PowerFill	2 mm	−	3 s	39.08 ± 2.78	67.03 ± 8.18	27.94 ± 7.78
Tetric PowerFill	2 mm	+	20 s	40.03 ± 2.89	65.93 ± 3.48	25.90 ± 3.96
Tetric PowerFill	2 mm	−	20 s	39.61 ± 4.19	71.92 ± 4.26	32.31 ± 5.96
Tetric PowerFill	4 mm	+	3 s	41.17 ± 3.63	68.77 ± 3.07	27.60 ± 6.14
Tetric PowerFill	4 mm	−	3 s	38.42 ± 1.24	64.86 ± 10.62	26.44 ± 11.76
Tetric PowerFill	4 mm	+	20 s	41.92 ± 2.74	66.65 ± 4.16	24.73 ± 5.04
Tetric PowerFill	4 mm	−	20 s	41.56 ± 1.55	70.72 ± 4.15	29.16 ± 4.64
IPS Empress Direct	2 mm	+	20 s	37.33 ± 2.53	78.24 ± 3.00	40.90 ± 5.00
IPS Empress Direct	2 mm	−	20 s	34.62 ± 1.06	74.33 ± 4.79	39.71 ± 4.28

+, glycerin applied; −, no glycerin applied. VHN, Vickers hardness number; SD, standard deviation. “Change after polishing” is the within-specimen difference (after − before polishing).

**Table 4:** Results of the surface roughness measurements (Ra, µm) for the bulk-fill resin composite as a function of polishing (yes/no), glycerin application (yes/no), layer thickness (2 mm/4 mm), and photoactivation protocol (3 s/20 s). Specimens were measured before and after polishing.

Variable	Category	β	95% CI Lower	95% CI Upper	*p*-Value
Intercept	—	0.54	0.46	0.62	—
Polishing	No	Ref.	—	—	—
	Yes	−0.47	−0.57	−0.38	<0.001
Glycerin	No	Ref.	—	—	—
	Yes	0.69	0.59	0.78	<0.001
Layer Thickness	2 mm	Ref.	—	—	—
	4 mm	0.03	−0.04	0.10	0.348
Photoactivation Protocol	3 s	Ref.	—	—	—
	20 s	0.05	−0.01	0.12	0.112
Polishing × Glycerin	Yes × Yes	−0.66	−0.80	−0.53	<0.001

Reference categories: no polishing, no glycerin application, 2 mm layer thickness, and 3 s high-speed photoactivation protocol. β values represent the estimated difference relative to the corresponding reference category. CI, confidence interval; Ref., reference category; —, not applicable.

**Table 5:** Results of the surface roughness measurements (Ra, µm) for the conventional resin composite as a function of glycerin application (yes/no) and polishing (yes/no). Specimens were measured before and after polishing.

Variable	Category	β	95% CI Lower	95% CI Upper	*p*-Value
Intercept	—	0.08	−0.12	0.27	—
Polishing	No	Ref.	—	—	—
	Yes	0.03	−0.26	0.32	0.824
Glycerin	No	Ref.	—	—	—
	Yes	1.69	1.41	1.97	<0.001
Polishing × Glycerin	Yes × Yes	−1.64	−2.06	−1.23	<0.001

Reference categories: no polishing and no glycerin application. β values represent the estimated difference relative to the corresponding reference category. CI, confidence interval; Ref., reference category; —, not applicable.

**Table 6:** Results of the surface microhardness measurements (VHNs) for the bulk-fill resin composite as a function of polishing (yes/no), glycerin application (yes/no), layer thickness (2 mm/4 mm), and photoactivation protocol (3 s/20 s). Specimens were measured before and after polishing.

Variable	Category	β	95% CI Lower	95% CI Upper	*p*-Value
Intercept	—	37.63	35.23	40.03	—
Polishing	No	Ref.	—	—	—
	Yes	28.97	26.02	31.91	<0.001
Glycerin	No	Ref.	—	—	—
	Yes	1.47	−1.33	4.27	0.301
Layer Thickness	2 mm	Ref.	—	—	—
	4 mm	1.51	−0.43	3.44	0.135
Photoactivation Protocol	3 s	Ref.	—	—	—
	20 s	2.58	0.64	4.51	0.012
Polishing × Glycerin	Yes × Yes	−5.52	−9.69	−1.36	0.007

Reference categories: no polishing, no glycerin application, 2 mm layer thickness, and 3 s high-speed photoactivation protocol. β values represent the estimated difference relative to the corresponding reference category. VHN, Vickers hardness number; CI, confidence interval; Ref., reference category; —, not applicable.

**Table 7:** Results of the surface microhardness measurements (VHNs) for the conventional resin composite as a function of glycerin application (yes/no) and polishing (yes/no). Specimens were measured before and after polishing.

Variable	Category	β	95% CI Lower	95% CI Upper	*p*-Value
Intercept	—	34.32	32.07	36.58	—
Polishing	No	Ref.	—	—	—
	Yes	40.31	37.46	43.15	<0.001
Glycerin	No	Ref.	—	—	—
	Yes	3.31	0.59	6.03	0.016

Reference categories: no polishing and no glycerin application. β values represent the estimated difference relative to the corresponding reference category. VHN, Vickers hardness number; CI, confidence interval; Ref., reference category; —, not applicable.

## Data Availability

The raw data supporting the conclusions of this article will be made available by the authors on request.

## Supplementary Materials

The following supporting information can be downloaded at: https://www.mdpi.com/article/10.3390/polym18182210/s1, Table S1: Initial full factorial models for each material and outcome, including all interaction terms, before backward elimination; Table S2: Direct between-material comparison under the experimental conditions common to both composites (2 mm thickness and conventional 20 s photoactivation); Table S3: Intermediate models for the bulk-fill composite, documenting the backward-elimination step for the higher-order interactions.
