# Supplementary material for: Beyond High-Speed Curing: Effects of Layer Thickness, Glycerin, and Polishing on the Surface Properties of an AFCT-Based Bulk-Fill Composite
Source: Polymers (Basel). 2026 Sep 11;18(18):2210. doi: 10.3390/polym18182210 (PMC13611165; doi:10.3390/polym18182210)
Supplement: Supplementary file 1 [file polymers-18-02210-s001.zip › polymers-4534224-supplementary.pdf]

Supplementary Materials

Beyond High-Speed Curing: Effects of Layer Thickness, Glycerin, and Polishing on the Surface Properties of an AFCT-Based Bulk-Fill Composite

Ezgi Erden Kayalidere, Merve Sahin and Zeynep Hale Keles

Table S1. Initial full factorial models for each material and outcome, including all interaction terms, before backward elimination.

Estimates are from the initial models described in Section 2.6, before backward elimination. For the bulk-fill composite the initial model contained the four main effects, all six two-way interactions, all four three-way interactions and the four-way interaction; for the conventional composite, in which only glycerin and polishing were varied, it contained both main effects and their interaction. Reference categories: no polishing, no glycerin, 2-mm layer thickness, 3-s high-speed photoactivation protocol. Main effects in the initial full factorial model are conditional on the reference levels of the interacting factors and are therefore not directly comparable with the corresponding main effects in the reduced models reported in Tables 4–7. The individual specimen was the experimental unit; each specimen contributes one value before and one value after polishing. Terms retained in the final models (Tables 4–7) are shown in bold.

**Term**	**β**	**95% CI lower**	**95% CI upper**	**p**
Tetric PowerFill — surface roughness (Ra, µm)				
**Intercept**	**0.460**	**0.331**	**0.590**	**<0.001**
**Polishing (yes)**	**−0.352**	**−0.537**	**−0.166**	**<0.001**
**Glycerin (yes)**	**0.809**	**0.626**	**0.992**	**<0.001**
**Layer thickness (4 mm)**	**0.203**	**0.020**	**0.386**	**0.030**
**Photoactivation protocol (20 s)**	**0.091**	**−0.092**	**0.274**	**0.326**
**Polishing × Glycerin**	**−0.782**	**−1.044**	**−0.520**	**<0.001**
Polishing × Layer thickness	−0.206	−0.468	0.055	0.119
Polishing × Photoactivation protocol	−0.075	−0.337	0.187	0.565
Glycerin × Layer thickness	−0.363	−0.622	−0.104	0.007
Glycerin × Photoactivation protocol	−0.074	−0.333	0.185	0.573
Layer thickness × Photoactivation protocol	−0.090	−0.349	0.169	0.491
Polishing × Glycerin × Layer thickness	0.369	−0.001	0.739	0.051
Polishing × Glycerin × Photoactivation protocol	0.066	−0.304	0.436	0.721
Polishing × Layer thickness × Photoactivation protocol	0.078	−0.292	0.448	0.673
Glycerin × Layer thickness × Photoactivation protocol	0.383	0.017	0.749	0.041

Polishing × Glycerin × Layer thickness × Photoactivation protocol	−0.396	−0.920	0.127	0.134
Tetric PowerFill — microhardness (VHN)				
**Intercept**	**39.08**	**35.40**	**42.76**	**<0.001**
**Polishing (yes)**	**27.94**	**22.53**	**33.36**	**<0.001**
**Glycerin (yes)**	**2.35**	**−2.86**	**7.56**	**0.372**
**Layer thickness (4 mm)**	**−0.66**	**−5.87**	**4.54**	**0.800**
**Photoactivation protocol (20 s)**	**0.53**	**−4.67**	**5.74**	**0.840**
**Polishing × Glycerin**	**−12.40**	**−20.07**	**−4.74**	**0.002**
Polishing × Layer thickness	−1.50	−9.16	6.16	0.695
Polishing × Photoactivation protocol	4.37	−3.30	12.03	0.256
Glycerin × Layer thickness	0.40	−6.96	7.76	0.914
Glycerin × Photoactivation protocol	−1.93	−9.30	5.43	0.603
Layer thickness × Photoactivation protocol	2.61	−4.76	9.97	0.483
Polishing × Glycerin × Layer thickness	13.56	2.72	24.39	0.015
Polishing × Glycerin × Photoactivation protocol	6.00	−4.84	16.83	0.270
Polishing × Layer thickness × Photoactivation protocol	−1.65	−12.48	9.19	0.760
Glycerin × Layer thickness × Photoactivation protocol	−0.46	−10.87	9.96	0.931
Polishing × Glycerin × Layer thickness × Photoactivation protocol	−11.58	−26.90	3.74	0.134
IPS Empress Direct — surface roughness (Ra, µm)				
**Intercept**	**0.075**	**−0.121**	**0.270**	**0.434**
**Polishing (yes)**	**0.030**	**−0.261**	**0.321**	**0.824**
**Glycerin (yes)**	**1.691**	**1.414**	**1.967**	**<0.001**

**Polishing × Glycerin**	**−1.644**	**−2.055**	**−1.232**	**<0.001**
IPS Empress Direct — microhardness (VHN)				
**Intercept**	**34.62**	**31.94**	**37.30**	**<0.001**
**Polishing (yes)**	**39.71**	**35.48**	**43.94**	**<0.001**
**Glycerin (yes)**	**2.71**	**−1.08**	**6.50**	**0.151**
Polishing × Glycerin	1.20	−4.79	7.18	0.666

Because the model was refitted after each elimination step, the statistical significance of individual terms could change as other terms were removed. The intermediate models at which the remaining higher-order interactions were evaluated are given in Table S3.

Table S2. Direct between-material comparison under the experimental conditions common to both compo-sites (2 mm thickness and conventional 20 s photoactivation).

Material and glycerin application were between-specimen factors and polishing the within-specimen repeated factor; the model included the main effects of material, glycerin, and polishing, and all corresponding two- and three-way interactions. The analysis comprised 24 specimens, 12 per material, each measured before and after polishing. Between-specimen terms are estimated from the specimen means and within-specimen terms from the within-specimen change (after − before polishing); both are tested on 20 degrees of freedom. Reference categories: Tetric PowerFill, no glycerin, before polishing.

**Term**	**β**	**95% CI lower**	**95% CI upper**	**p**
Surface roughness (Ra, μm) — between-specimen effects				
Intercept	0.338	0.215	0.461	<0.001
Material (IPS Empress Direct)	−0.248	−0.423	−0.074	0.008
Glycerin (yes)	0.377	0.203	0.552	<0.001
Glycerin × Material	0.491	0.245	0.738	<0.001
Surface roughness (Ra, μm) — within-specimen effects				
Polishing	−0.427	−0.665	−0.188	0.001
Polishing × Material	0.456	0.119	0.794	0.011
Polishing × Glycerin	−0.716	−1.054	−0.379	<0.001
Polishing × Glycerin × Material	−0.928	−1.405	−0.451	<0.001
Microhardness (VHN) — between-specimen effects				
Intercept	55.77	53.67	57.86	<0.001
Material (IPS Empress Direct)	−1.29	−4.26	1.67	0.374
Glycerin (yes)	−2.79	−5.75	0.17	0.064
Glycerin × Material	6.10	1.91	10.29	0.007
Microhardness (VHN) — within-specimen effects				
Polishing	32.31	28.17	36.45	<0.001
Polishing × Material	7.40	1.55	13.25	0.016

Polishing × Glycerin	−6.41	−12.26	−0.56	0.033
Polishing × Glycerin × Material	7.60	−0.67	15.88	0.070

For both outcomes the glycerin × material and polishing × material interactions are significant, showing that the effects of glycerin and of polishing differ between the two composites under identical experimental conditions. The polishing × glycerin × material interaction is significant for roughness but not for microhardness ($p = 0.070$), so for microhardness the present data do not establish that the material difference varies with polishing status.

Table S3. Intermediate models for the bulk-fill composite, documenting the backward-elimination step for the higher-order interactions.

These models correspond to the elimination step at which the remaining higher-order interactions were evaluated. As in Table S2, between-specimen terms are estimated from the specimen means and within-specimen terms from the within-specimen change (after − before polishing), so that each effect is tested against the appropriate error term; interactions involving polishing therefore appear among the within-specimen effects. Reference categories are as in Table S1, but because the two tables report different models, individual coefficients are not directly comparable between them. The conventional composite is not shown because its model was unchanged between the initial and the final stage.

Term	β	95% CI lower	95% CI upper
**Tetric PowerFill — surface roughness (Ra, μm): between-specimen effects**			
Intercept	0.300	0.225	0.375
Glycerin (yes)	0.398	0.306	0.489
Layer thickness (4 mm)	0.074	−0.017	0.166
Photoactivation protocol (20 s)	0.023	−0.052	0.098
Glycerin × Layer thickness	−0.147	−0.297	0.002
**Glycerin × Layer thickness × Photoactivation protocol**	**0.123**	**−0.026**	**0.273**
**Tetric PowerFill — surface roughness (Ra, μm): within-specimen effects**			
Polishing (yes)	−0.389	−0.523	−0.255
Polishing × Glycerin	−0.749	−0.939	−0.559
Polishing × Layer thickness	−0.167	−0.357	0.023
Polishing × Glycerin × Layer thickness	0.171	−0.098	0.440
**Tetric PowerFill — microhardness (VHN): between-specimen effects**			
Intercept	52.11	50.17	54.05
Glycerin (yes)	−1.29	−3.23	0.64
Layer thickness (4 mm)	1.51	−0.43	3.44
Photoactivation protocol (20 s)	2.58	0.64	4.51
**Tetric PowerFill — microhardness (VHN): within-specimen effects**			
Polishing (yes)	30.13	26.06	34.19
Polishing × Glycerin	−9.41	−15.16	−3.66
Polishing × Layer thickness	−2.32	−8.07	3.43
**Polishing × Glycerin × Layer thickness**	**7.77**	**−0.37**	**15.90**

The two terms shown in bold are those whose p-values were below 0.05 in the initial models (Table S1): glycerin × layer thickness × photoactivation protocol for roughness ($p = 0.041$) and polishing × glycerin × layer thickness for microhardness ($p = 0.015$). At this step the 95% confidence interval of each includes zero, and both were accordingly removed. The polishing × glycerin × layer thickness interaction for roughness ($p = 0.051$ in the initial model) was carried forward to this step, where its interval also includes zero, and was removed at the next; retaining it in the final model changes neither the direction nor the significance of any effect in Table 4. Glycerin × layer thickness is shown here with the remaining higher-order terms still in the model; once those were removed it was no longer significant ($p = 0.198$), as reported in the main text.
